# Abdominal Lymphonodular Cryptococcosis in an Immunocompetent Child

**DOI:** 10.1155/2015/347403

**Published:** 2015-11-16

**Authors:** Mehjabeen Zaidi, Sonia Qureshi, Sadia Shakoor, Saira Fatima, Fatima Mir

**Affiliations:** ^1^Department of Pediatrics and Child Health, Aga Khan University, Stadium Road, Karachi 74800, Pakistan; ^2^Microbiology, Department of Pathology, Aga Khan University, Stadium Road, Karachi 74800, Pakistan; ^3^Histopathology, Department of Pathology, Aga Khan University, Stadium Road, Karachi 74800, Pakistan

## Abstract

We describe our experience with an apparently immunocompetent child presenting with pyrexia of unknown origin without focal signs. Investigations revealed lymphadenopathy at lung hila, mesentery, and porta hepatis. The child had received at least two months of empiric antituberculous therapy (ATT) before she came to us. A CT-guided biopsy revealed granulomatous inflammation. PAS stain showed yeasts which stained blue with Alcian blue, suggesting* C. neoformans*.

## 1. Background

Lymphonodular cryptococcosis has been reported in the past in immunocompromised and immunocompetent children [1, 2]. The two species,* C. neoformans* and* C. gattii,* enter the respiratory system through inhalation and disseminate hematogenously to various sites in the body [3]. Disease manifestations range from localized disease such as meningitis, pulmonary infiltrates, and lymphadenopathy to disseminated cryptococcosis with multiorgan involvement [3]. Cryptococcosis has classically been associated with acquired and inherited T-cell deficiencies [4]. Though antiretroviral therapy has decreased the incidence of cryptococcosis in HIV patients, it is increasingly being reported in a non-HIV, nontransplant cohort [5]. The underlying pathophysiology is not clear. We report the case history of a 5-year-old female child with Fever of Unknown Origin (FUO) and abdominal lymphadenopathy. A CT-guided lymph node biopsy showed granulomatous inflammation and encapsulated yeasts resembling* Cryptococcus* species. Culture was negative for Acid Fast Bacillus (AFB) and bacterial and fungal cultures could not be sent due to inadequate tissue sample. The child responded clinically and serologically to 6 months of oral fluconazole and remains well to date.

## 2. Case History

A 5-year-old female child had remained well and thriving till 10 weeks before presentation when she developed high grade, continuous fever associated with nonradiating, central abdominal pain and anorexia. She had started taking three-drug antituberculous therapy (ATT) (isoniazid, rifampicin, and pyrazinamide) based on a general practitioner's prescription/physician discretion due to the clinical finding of nontender lymphadenopathy and unabated fever for 2 weeks. The modified Keith Edwards scoring had not been done and neither had the tissue culture been sought. By week 8 of empiric ATT for presumed TB adenitis, there was no clinical improvement and a weight loss of 2.5 kg was observed.

She was the second amongst 3 siblings and a student of kindergarten. She had a positive BCG scar with immunization complete for age as per Pakistan's Expanded Programme of Immunization (EPI) schedule. There was no contact history for tuberculosis (TB), no family pets, or travel beyond her hometown. She belonged to a middle-income family and was a resident of a neighbouring city (approximately 160 km away from Karachi). The father was a civil servant, and the mother was a housewife.

On examination, the child was febrile, icteric, and pale with hepatomegaly. There was no peripheral adenopathy.

Investigations done prior to presentation in the Infectious Disease (ID) clinic revealed normochromic, normocytic anemia (Hb 7.4 mg/dL), a progressively rising white count over twelve weeks of fever (21 × 10^9^/L at outset to 32 × 10^9^/L at presentation in ID clinic), high inflammatory markers: Erythrocyte Sedimentation Rate (ESR 135 mm/hr) and C-reactive protein (CRP29), direct hyperbilirubinemia (total bilirubin 4.6 with 3.7 direct component), and an episode of* E. coli *Urinary Tract Infection (UTI), along with lymphadenopathy seen at the lung hila (chest X-Ray), mesentery, and porta hepatis 3.9 × 7 cm in AP and transverse dimension in the mesentery and 4 × 5 cm AP and transverse dimension at the porta hepatis (CT abdomen).

Tests conducted in the ID clinic as workup for “pyrexia of unknown origin” were negative for malaria, typhoid (blood and bone marrow culture), malignancy (bone marrow smear and trephine results showing normal erythropoiesis, myelopoiesis, and megakaryocytes), and autoimmune disease (negative dsDNA, rheumatoid factor, and Coombs test).

A chest X-ray and abdominal ultrasound were repeated to monitor size of lymph nodes after 2 months of ATT. They remained as before. Tissue diagnosis was stressed on and a CT-guided mesenteric lymph node biopsy was carried out and sent for histopathology and an AFB culture, keeping our top differentials of malignancy and multidrug resistant tuberculosis (MDR-TB) in mind. Fungal and bacterial cultures could not be sent due to inadequate tissue.

**Figure 1:**
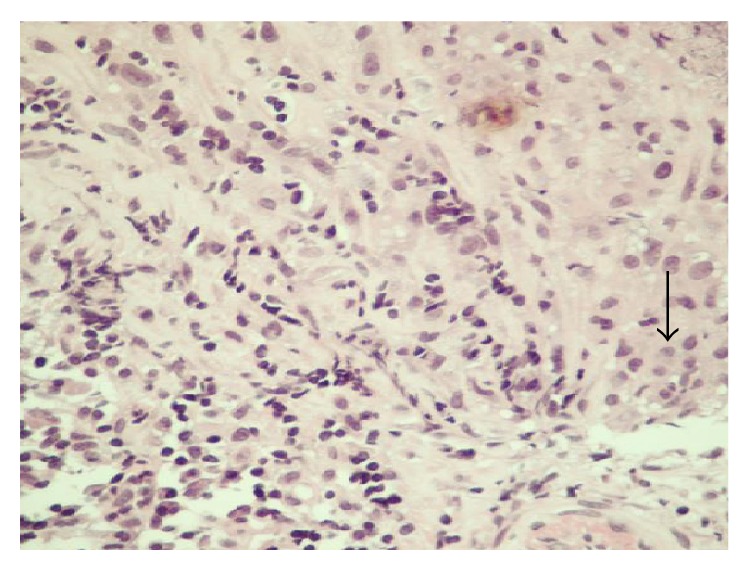
H&E stain of mesenteric lymph node biopsy showing granuloma formation, 400x.

**Figure 2:**
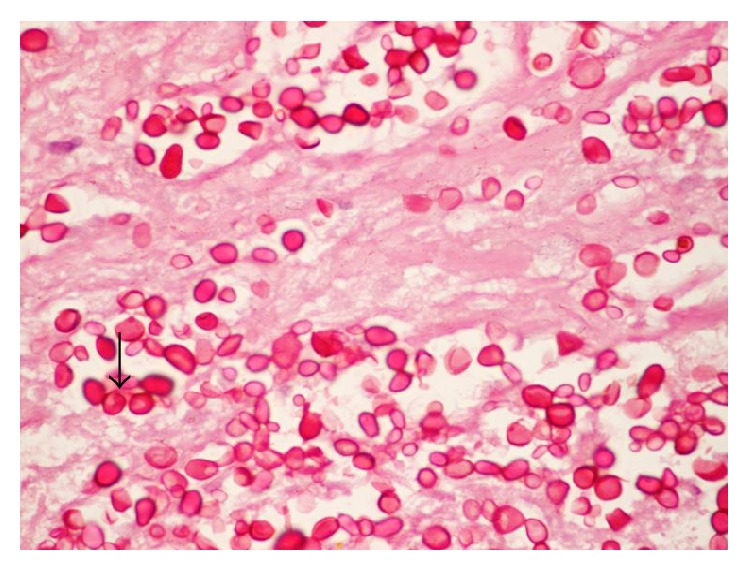
PAS stain of mesenteric lymph node biopsy showing yeasts, 1000x.

**Figure 3:**
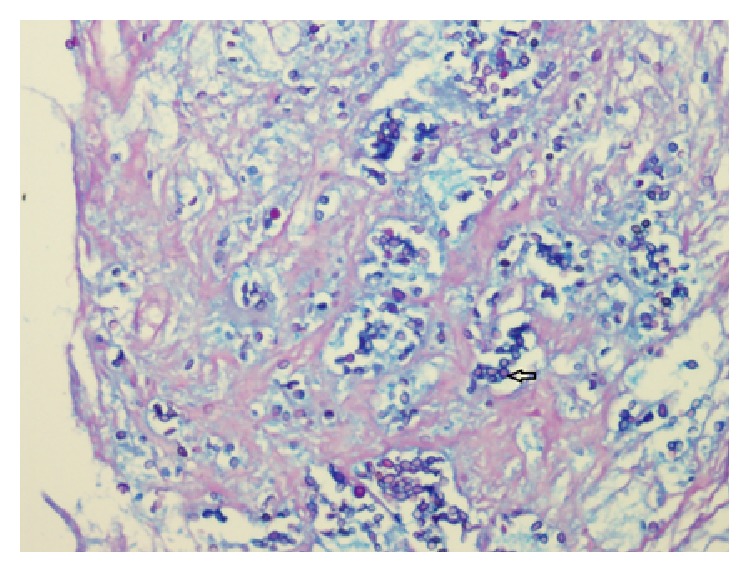
PAS stain with Alcian blue, 1000x.

Histopathology sections revealed extensive necrosis with aggregates of epithelioid histiocytes forming granulomas in the core tissue (see arrow in Figure 1). Few Langhans-type giant cells were also seen. Periodic acid-Schiff (PAS) stain showed yeasts (see Figure 2) and capsules stained blue with Alcian blue (arrow Figure 3) suggesting* C. neoformans*.

Additional tests done after the biopsy result revealed a very high cryptococcal antigen titre (>1 : 1024) and a negative HIV antibody. Though the underlying T-cell deficiency was considered, quantitative evaluation (flow cytometry) was deferred because of the absence of a clinical history suggesting immune compromise and also because cryptococcosis is not exclusively an immunocompromised-host disease. The parents refused lumbar puncture because of scepticism of occult CNS disease in the absence of signs and symptoms. The child was assumed to have high antigenemia with lymph node involvement and unknown CNS status. Amphotericin was started at a dose of 0.5 mg/kg/day and increased over 2-3 days to 1.5 mg/kg/day. Flucytosine, recommended by the IDSA in conjunction with amphotericin for cryptococcosis, was not available in Karachi. The child was ultimately discharged home on oral fluconazole monotherapy (6 mg/kg/day) as the parents were reluctant to administer prolonged parenteral amphotericin at home.

At one-month follow-up, the patient had gained 2 kg, had defervesced, and resumed normal home and school activities. At 3 months, she was clinically well with improvement in lymph node size on abdominal ultrasound and chest X-ray. Therapy was monitored by repeat cryptococcal antigen titres which progressively decreased over 6 months of therapy (more than 1 : 1024 during admission, 1 : 1024 one month, 1 : 256 three months, and 1 : 32 five months of therapy). The family did not return to our center for a follow-up or immune workup beyond 6 months. The patient is doing well after cessation of therapy and continues to thrive as per telephonic contact with parents.

## 3. Discussion

Pathogenesis of cryptococcosis in “phenotypically normal” hosts like our patient, without predisposing factors such as HIV, transplant, clinical history suggesting T-cell immunodeficiency, or chronic conditions such as end-stage liver disease and renal insufficiency, has been studied [4]. It involves innate (macrophages, complement and Natural Killer) (NK) defense mechanisms, and cell-mediated (CD4 cells) and humoral immunity (facilitation of phagocytosis) [6]. Pathogen factors such as the capsule and host factors such as surfactant at the alveolar level have been implicated in helping the organism evade detection by macrophages. Although precisely how the human host controls cryptococci remains unclear, clinical practice suggests that immunosuppressive agents like corticosteroids allow cryptococci to reactivate, CD4 cell numbers are critically important to host immunity, and cryptococcal proliferation in humans is clearly contained by a vigorous granulomatous response [7, 8]. Cryptococcosis should therefore be considered as a differential in the workup for PUO, especially in the presence of granulomatous inflammation.

Our patient had an uneventful first 5 years of life till the onset of this illness. Changing trends in epidemiology of cryptococcosis in China shows that the majority of cases occurred in a non-HIV, nontransplant “phenotypically normal” cohort [5]. Kiertiburanakul et al. in a retrospective review (1987–2003) of HIV-negative patients with positive cryptococcal cultures showed that 35% patients had no underlying conditions [9]. Idiopathic CD4 lymphocytopenia (ICL) was found in 11 patients with cryptococcosis reviewed over a 12-month period with features similar to those of cryptococcal infections in normal hosts [10]. Our threshold for suspecting immunodeficiency was very high. Ideally quantitative deficiencies, especially in the absence of the availability of qualitative immune tests in Karachi, would have been useful in deciding on the drug of choice, duration of treatment, and prognostication. The family was retrospectively counseled to get an immune workup in case of prolonged or unusual fever.

CNS is the commonest system affected in cryptococcosis [3]. Occult disease can occur without overt signs and symptoms. Though the parents refused a lumbar puncture, they were extensively counseled that this would mean a longer course of therapy and difficulty in prognostication. Pulmonary involvement has been reported in 10–36% of non-HIV patients [11]. Symptoms can be absent in up to one-third of the patients with abnormal X-rays. Our child had no pulmonary symptoms: her chest X-ray showed normal lung fields and hilar lymphadenopathy.

Tissue-based PCR is the gold standard for diagnosis on histopathology [12]. This was not done as it was unavailable in our institution. Liaw et al. report direct determination of cryptococcal antigen through latex agglutination system in transthoracic needle lung aspirate with a positive predictive value of 89% and a negative predictive value of 100% [13]. Fridlington et al. have also reported using Tzanck smear on skin biopsy which revealed narrow-based budding encapsulated yeasts, suggesting cryptococcosis, and was confirmed by fungal culture [14]. These may have additive value in the absence of tissue-PCR and fungal culture.

Cryptococcal lymphadenopathy has been reported in HIV and immunocompetent patients with some and apparently no immune deficiency [1, 15–17]. Karagüzel reported a child with mesenteric cryptococcal lymphadenitis who presented with acute abdomen [18].

Wu et al. reported on a 10-year-old boy with disseminated cryptococcosis resembling lymphoreticular malignancy [19]. Differentials of granulomatous inflammation on biopsy in countries like Pakistan should include cryptococcosis.

IDSA guidelines recommend that cryptococcemia or dissemination (involvement of at least 2 noncontiguous sites or evidence of high fungal burden based on cryptococcal antigen titer 1 : 512), as in our case, should be treated as CNS disease (2 weeks of induction therapy with IV amphotericin with flucytosine, followed by 8 weeks of maintenance therapy with oral fluconazole) [20]. Our patient received IV amphotericin while in hospital (approximately 3 days between histopathology reporting and discharge). The family refused parenteral therapy even though they were counseled about the drug of choice in the absence of ruling out CNS involvement. The patient was sent home on oral fluconazole therapy for 6–12 months with instructions to have cryptococcal antigen titre repeated every 2 months. She remained lost to follow-up for almost two years after 6-month therapy until the parents contacted the primary physician. The child remains well and asymptomatic to date. Induction with fluconazole has been recommended only for patients with no antigenemia and no CNS disease and only single site involvement. Our patient had very high antigenemia and lymphadenopathy (mesenteric and porta hepatis, the latter causing secondary cholestasis).

Heteroresistance and emerging resistance have been implicated as possible causes in reactivation of cryptococcosis in patients treated with fluconazole in induction phase [21].

We were relatively successful in monitoring therapy success with the help of progressively falling antigen titres over 6 months of therapy. However, patients like these should be ideally followed posttreatment and investigated for immune deficiency.

We should also send tissue for fungal and AFB cultures in addition to bacterial cultures when faced with lymphadenopathy and Fever of Unknown Origin, or empiric ATT failures.

## 4. Conclusion

Cryptococcosis should be considered as a differential of Fever of Unknown Origin and granulomatous lymphadenopathy in Pakistan. Patients should be investigated for immunodeficiency, and extent of disease should be established. Clinical suspicion must be communicated at the time of sending specimens to the laboratory so that optimal diagnostic tools can be resourced.
